# High-Throughput Accurate Single-Cell Screening of *Euglena gracilis* with Fluorescence-Assisted Optofluidic Time-Stretch Microscopy

**DOI:** 10.1371/journal.pone.0166214

**Published:** 2016-11-15

**Authors:** Baoshan Guo, Cheng Lei, Takuro Ito, Yiyue Jiang, Yasuyuki Ozeki, Keisuke Goda

**Affiliations:** 1 Department of Chemistry, University of Tokyo, Tokyo 113–0033, Japan; 2 Department of Electronic Engineering, Tsinghua University, Beijing 100084, China; 3 Japan Science and Technology Agency, Kawaguchi 332–0012, Japan; 4 Department of Electrical Engineering and Information Systems, University of Tokyo, Tokyo 113–8656, Japan; 5 Department of Electrical Engineering, University of California Los Angeles, Los Angeles, California, 90095, United States of America; Institute of Human Virology, UNITED STATES

## Abstract

The development of reliable, sustainable, and economical sources of alternative fuels is an important, but challenging goal for the world. As an alternative to liquid fossil fuels, algal biofuel is expected to play a key role in alleviating global warming since algae absorb atmospheric CO_2_ via photosynthesis. Among various algae for fuel production, *Euglena gracilis* is an attractive microalgal species as it is known to produce wax ester (good for biodiesel and aviation fuel) within lipid droplets. To date, while there exist many techniques for inducing microalgal cells to produce and accumulate lipid with high efficiency, few analytical methods are available for characterizing a population of such lipid-accumulated microalgae including *E*. *gracilis* with high throughout, high accuracy, and single-cell resolution simultaneously. Here we demonstrate high-throughput, high-accuracy, single-cell screening of *E*. *gracilis* with fluorescence-assisted optofluidic time-stretch microscopy–a method that combines the strengths of microfluidic cell focusing, optical time-stretch microscopy, and fluorescence detection used in conventional flow cytometry. Specifically, our fluorescence-assisted optofluidic time-stretch microscope consists of an optical time-stretch microscope and a fluorescence analyzer on top of a hydrodynamically focusing microfluidic device and can detect fluorescence from every *E*. *gracilis* cell in a population and simultaneously obtain its image with a high throughput of 10,000 cells/s. With the multi-dimensional information acquired by the system, we classify nitrogen-sufficient (ordinary) and nitrogen-deficient (lipid-accumulated) *E*. *gracilis* cells with a low false positive rate of 1.0%. This method holds promise for evaluating cultivation techniques and selective breeding for microalgae-based biofuel production.

## Introduction

Due to a limited supply of fossil fuels and the dangers of increasing atmospheric CO_2_ levels, the development of reliable, sustainable, and economical sources of alternative fuels is an important, but challenging goal for the world [1]. As an alternative to liquid fossil fuels, algal biofuel has attracted much attention from the scientific community over the last decade. It is based on algae as a source of energy-rich hydrocarbon and expected to be a potential solution to global warming since algae absorb atmospheric CO_2_ via photosynthesis [2,3]–the only process that can convert CO_2_ into organic compounds with high energy content and can, hence, provide a source for sustainable transport fuel production. Furthermore, algae require minimal environmental resources because they can be grown even in saline and wastewater. Among various types of algae for biofuel production, *Euglena gracilis*, a species of unicellular flagellate protists found in fresh water, is attractive as it is known to produce wax ester within lipid droplets (which can be refined to produce kerosene suitable as an aviation fuel) [3,4].

To date, several cultivation techniques for inducing the accumulation of intracellular lipid have been developed to increase the efficiency of biofuel production in microalgae such as *E*. *gracilis* [5–7]. In general, microalgae can alter their lipid metabolism in response to changes in environmental conditions [8,9]. Under stress conditions such as nutrient starvation, UV irradiation, and anaerobicity, many types of microalgae modify their lipid biosynthetic pathways toward the formation and accumulation of neutral lipids, mainly in the form of wax ester and/or triacylglycerol such that microalgae can endure these adverse conditions [9]. Among such techniques for lipid induction, the most widely used technique is nutrient starvation, in particular nitrogen deficiency. Nitrogen is a primary growth-limiting factor for eukaryotic microalgae (e.g., *E*. *gracilis*) and is one of the first nutrients to be depleted during algal cultivation [10]. It has been reported that nitrogen deficiency in microalgae not only affects its fatty acid metabolism, but also affects its pigment composition [11,12].

Despite the availability of such lipid-inducing cultivation techniques, their effect on a heterogeneous population of microalgal cells has not been fully explored and exploited due to the lack of analytical tools that allow rapid interference-free or non-invasive evaluation of a large number of cells at single-cell resolution. Traditionally, flow cytometry has been used as a standard tool for large-scale cell analysis by virtue of its capability of a rapid, objective, and sensitive evaluation of a large population of individual cells [13,14], but its operation is based on single-point light scattering and fluorescence detection and does not have spatial metrics to characterize morphological and intracellular phenotypes of cells. While imaging flow cytometry, a relatively new type of flow cytometry equipped with an imaging capability, offers rich information about individual cells and can distinguish clustered cells and debris that would otherwise result in false positive events in conventional flow cytometry, its throughput is constrained to ~1,000 cells/s due to the limited frame rate of its build-in image sensor [15]. For high-accuracy screening of microalgal cells under different culture conditions, both high throughout and high content are required simultaneously.

**Fig 1:**
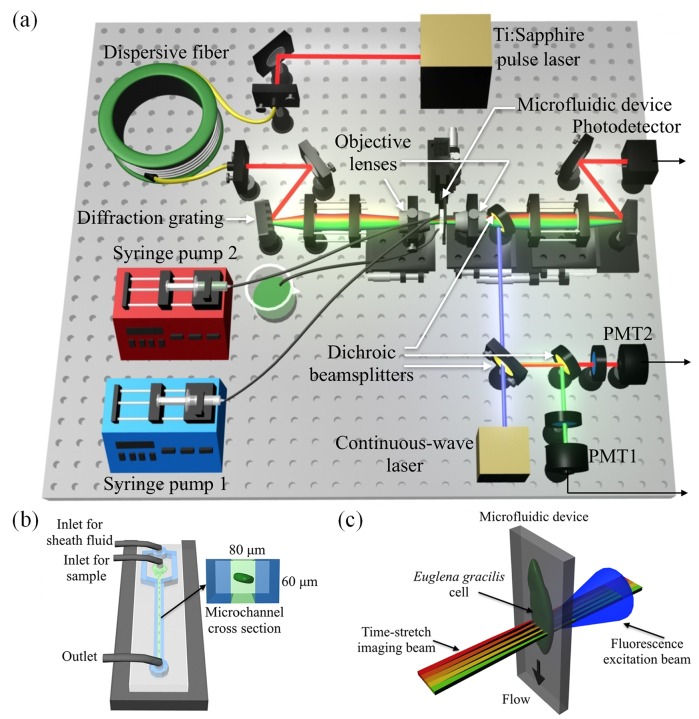
Optofluidic time-stretch microscope. (a) Schematic of the microscope. It consists of an optical time-stretch microscope, a two-channel fluorescence analyzer, a hydrodynamically focusing microfluidic device. The optical time-stretch microscope can image every single cell at a high throughput of 10,000 cells/s while the fluorescence analyzer can simultaneously detect fluorescence from each cell. (b) Schematic of the microfluidic device. (c) Enlarged view of the optical interrogation.

In this Research Article, to meet the above need, we demonstrate high-throughput, high-accuracy, single-cell screening of *E*. *gracilis* with fluorescence-assisted optofluidic time-stretch microscopy–a method that combines the strengths of microfluidic cell focusing, optical time-stretch microscopy, and fluorescence detection used in conventional flow cytometry. Our fluorescence-assisted optofluidic time-stretch microscope consists of an optical time-stretch microscope [16–23] and a two-channel fluorescence analyzer on top of a hydrodynamically focusing microfluidic device (Fig 1) and can simultaneously detect fluorescence from and obtain an image of every *E*. *gracilis* cell in a population with a high throughput of 10,000 cells/s. With the multi-dimensional information acquired by the system, we can classify nitrogen-sufficient (ordinary) and nitrogen-deficient (lipid-accumulated) *E*. *gracilis* cells with a false positive rate of 1.0%, which is 4 times less erroneous than traditional fluorescence-detection-based flow cytometry without sacrificing throughput. This method holds promise for evaluating the efficiency of lipid-inducing techniques as well as efficient selective breeding for microalgae-based biofuel production.

## Materials and Methods

### Optical time-stretch microscope

The optical time-stretch microscope is schematically shown in Fig 1A and similar to the microscope in our previous reports [22,23]. The optical source is a Ti:Sapphire femtosecond pulse laser (Tsunami, Spectra-Physics) with a center wavelength, bandwidth, and pulse repetition rate of 780 nm, 40 nm, and 75 MHz (corresponding to the 1D frame rate of the optical time-stretch microscope), respectively. A pulse from the laser enters a single-mode dispersive fibre spool with a group-velocity dispersion of -120 ps/nm (Nufern 630-HP) in which the pulse is temporally stretched by the dispersion. The time-stretched pulse is spatially dispersed by the first diffraction grating with a groove density of 1200 lines/mm and focused on the microchannel in the microfluidic device (Fig 1B) via the first objective lens (Olympus) with a magnification of 40× and a numerical aperture of 0.6. The transmitted pulse from the object (cell) in the microchannel is collected by the second objective lens with the same magnification and numerical aperture as the first objective lens and spatially recombined by the second diffraction grating identical in groove density to the first diffraction grating. Finally, the recombined pulse is detected by the high-speed photodetector (New Focus 1580-B) with a detection bandwidth of 12 GHz and digitized by the high-speed oscilloscope (Tektronix DPO 71604B) with a detection bandwidth of 16 GHz and a sampling rate of 50 GS/s. The oscilloscope’s signal recording is triggered by the autofluorescence detection events discussed below. Pulses are repeated at the repetition rate of 75 MHz while *E*. *gracilis* cells are flowing in the microchannel. During this process, the 1D cross-sectional profile of a flowing *E*. *gracilis* cell in the lateral direction (orthogonal to the flow direction) is encoded into the spectrum (i.e., the temporal waveform) of each detected pulse, such that the 2D spatial profile of the *E*. *gracilis* cell can be obtained by digitally stacking the 1D cross-sectional profiles (as shown in Fig 1C). Since the constructed images contain a 1D pattern reflecting the spectral structure of the laser source (usually Gaussian-shaped with ripples), we carefully removed the pattern to obtain the images of *E*. *gracilis* cells with a clear background. Finally, the classical dilation and erosion algorithm [24] is applied to the images in order to remove the background and leave only the effective parts of the *E*. *gracilis* cells. The spatial resolution of the optical time-stretch microscope was determined to be 780 nm in the imaging plane by performing a standard analysis of Rayleigh’s criterion using a USAF1951 resolution chart [22].

### Fluorescence analyzer

As shown in Fig 1A, the fluorescence analyzer is composed of a continuous-wave laser at 488 nm (OBIS 488LX, Coherent) which serves as an excitation light source, three dichroic beamsplitters, two photomultiplier tubes (PMTs) (Hamamatsu Photonics), and two band-pass filters with a bandwidth of 50 nm and a center pass wavelength of 526 nm and 690 nm, respectively. The excitation laser is combined with the optical time-stretch microscope via one of the dichroic beamsplitters, aligned with the light beam of the microscope, and focused onto flowing cells in the microfluidic device’s microchannel to excite intracellular chlorophyll and fluorescent dyes that label target molecules. The spot size of the laser light is much less than ~5 μm so that it probes a particular belt of the cell body during the flow. The excitation power at the flowing cells is about 30 mW. Two PMTs are used for detecting fluorescence whose spectral band is centered at 526 nm (PMT1) and for detecting fluorescence whose spectral band is centered at 690 nm (PMT2). As discussed below, PMT1 detects fluorescence emitted from a fluorescent dye that labels intracellular lipid droplets while PMT2 detects autofluorescence emitted from intracellular chlorophyll.

### Microfluidic device

For high-throughput single-cell imaging, we used a hydrodynamically focusing microfluidic device (shown in Fig 1B) that can focus and order every single fast-flowing *E*. *gracilis* cell. The device was fabricated using standard soft lithography methods [25]. The designed pattern was printed as a film mask (UnnoGiken). A negative photoresist (KMPR 1035, MicroChem) was spin-coated on a silicon wafer. The wafer was heated on a hot plate at 100°C for 10 min, exposed to UV light, baked again at 100°C for 5 min, developed using a SU-8 developer (MicroChem), washed with isopropyl alcohol and water, and heated again at 150°C for 15 min. Finally, the height of the mold was measured with a Dektak stylus profiler (Bruker Corp.). The negative photoresist mold on the silicon wafer was put in a petri dish and filled with PDMS (polydimethylsiloxane, Sylgard 184, Dow Corning) base and crosslinker at a 10:1 ratio. The petri dish was heated in an oven at 80°C for at least 1 hour. The PDMS layer was taken off from the mold while holes were punched through the PDMS for creating inlets and outlets. The device and slide glass were then treated with a plasma cleaner (Harrick Plasma). After the treatment, the device was permanently bonded to a glass slide and heated on a hot plate at 110°C for at least 10 min. The dimensions of the microchannel are 80 μm (width) and 60 μm (height). The device was connected with PEEK tubings (Upchurch Scientific) to two syringes (Thermo). The flow in the microchannel was driven by two syringe pump (Harvard Pump 11 Elite Infusion Models 70–450), one of which is for the sheath fluid at 400 μL/min and the other of which is for cells at 60 μL/min, resulting in a flow speed of 1.4 m/s in the microchannel. Based on the cell concentration of about 1.0 × 10^7^ cells/mL, the throughput was estimated to be approximately 10,000 cells/s.

### Preparation of *E*. *gracilis* cells

For the screening experiment below, we used *E*. *gracilis* NIES-48 provided by the Microbial Culture Collection at the National Institute for Environmental Studies (NIES, Japan). The cultures were grown in culture flasks with each working volume of 20 mL under 14/10 light/dark cycle illumination (130–150 μmol/m^2^/s) at 25°C. For all examinations, the stock culture of *E*. *gracilis* was grown in an autotrophic medium, AF-6 [26], as a pre-culture for at least 6 days. In their exponential growth phase, cells in AF-6 medium are under a nitrogen-sufficient condition whereas cells in AF-6‒N medium (nitrogen nutrient omitted from AF-6) are under a nitrogen-deficient condition after 5 days of cultivation (representing lipid-accumulated *E*. *gracilis* cells).

### Nitrogen deficiency for lipid accumulation in *E*. *gracilis* cells

It is known that *E*. *gracilis* accumulates paramylon, which is β-1,3-glucan in a crystalline form, as a reserve polysaccharide under nitrogen deficiency and converts it into a wax ester (lipid) which consists of a fatty acid, myristic acid, and myristyl alcohol to obtain energy in hypoxic conditions and can be utilized as an aviation fuel [27–29]. In our experiments below, we used both nitrogen-sufficient and nitrogen-deficient *E*. *gracilis* cells which contain little and much lipid contents, respectively. Since paramylon and lipids have different optical characteristics such as absorptivity and scattering cross section from other cytoplasmic components in *E*. *gracilis*, they can be used to distinguish differently cultivated *E*. *gracilis* cells by fluorescence-activated cell sorting (FACS) [27] and image cytometry [23].

### Staining of *E*. *gracilis* cells

*E*. *gracilis* cells were stained with a fluorescence dye called BODIPY 505/515 before the screening experiment below. The process of staining cells with BODIPY includes several steps. First, we dissolved BODIPY dyes in dimethyl sulfoxide (DMSO) to a concentration of 1 mM. Then, we mixed the BODIPY solution and *E*. *gracilis* cell solution in a ratio of 1:1. After incubating the mixed solution for 5 min, we washed the stained cells with DI water twice to remove residual BODIPY dyes in the solution. Finally, we obtained a cell concentration of 1.0 × 10^7^ cells/mL by adding a proper amount of DI water to the solution. The BODIPY dyes were attached to intracellular lipid in the *E*. *gracilis* cells and have a fluorescence emission spectrum centered at about 515 nm. If the cells do not contain lipid, BODIPY does not bind to intracellular lipid and is washed out through the washing process. Therefore, we can differentiate lipid-accumulated *E*. *gracilis* cells from ordinary cells by evaluating whether they have the fluorescence signal generated from their intracellular lipid.

## Results

### Simultaneous fluorescence detection and image acquisition

**Fig 2:**
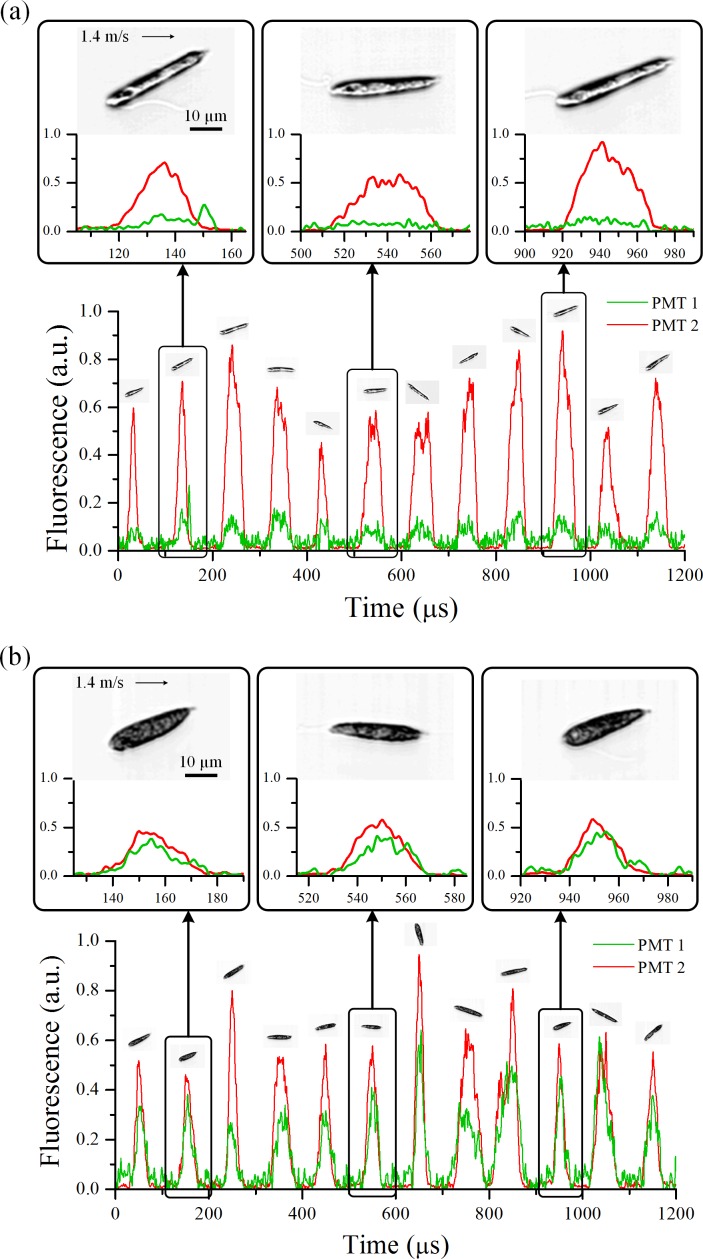
Simultaneous imaging and fluorescence-based characterization of single *E*. *gracilis* cells under two different culture conditions. Shown in the figure panels are a splice of multiple recorded waveforms triggered by autofluorescence detection events under each culture condition. (a) Nitrogen-sufficient cells. The images show that the cells are mostly transparent. The fluorescence signals indicate that the chlorophyll-oriented autofluorescence is strong while the BODIPY-tagged lipid fluorescence is weak. (b) Nitrogen-deficient cells. The images show that the cells are mostly opaque. The fluorescence signals indicate that both the chlorophyll-oriented autofluorescence and BODIPY-tagged lipid fluorescence are strong.

**Fig 3:**
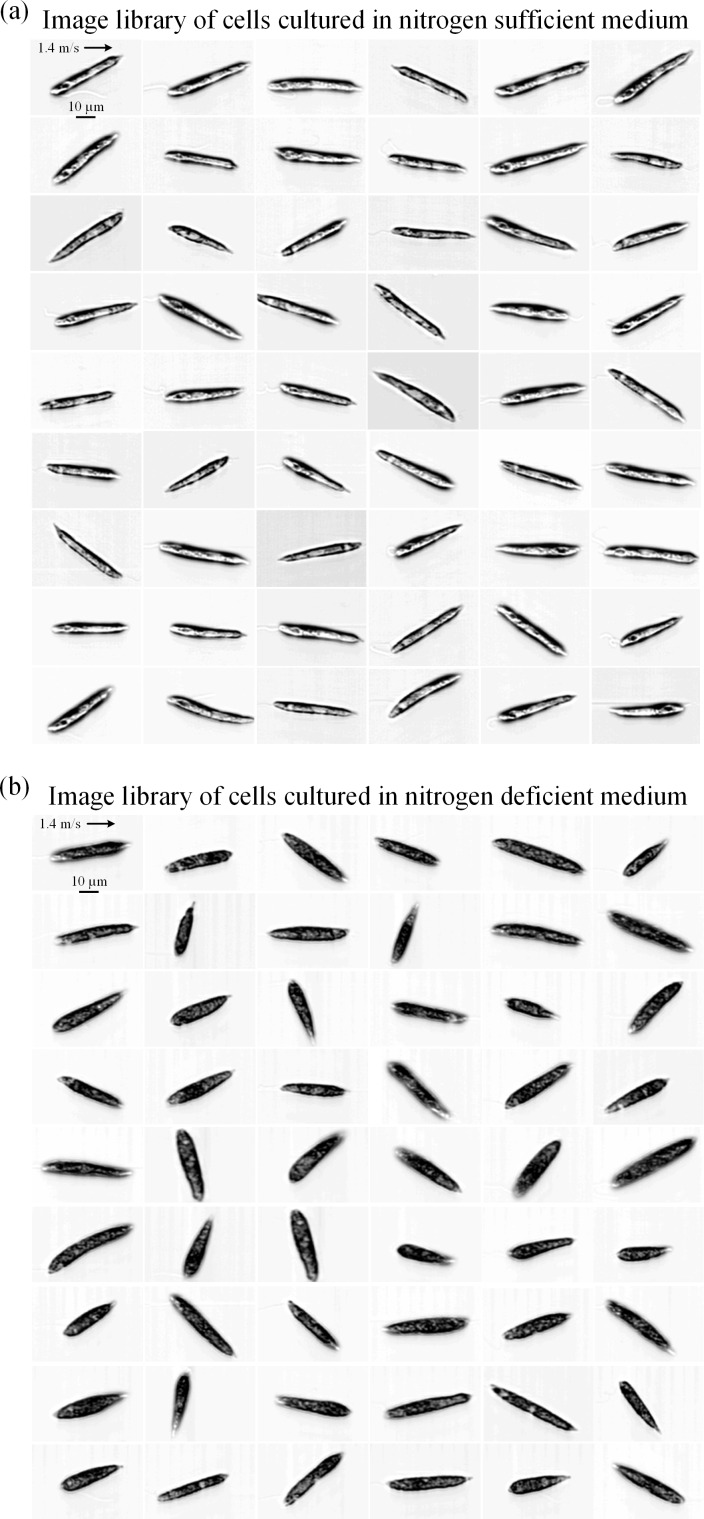
Images of *E*. *gracilis* cells under two different culture conditions. (a) Nitrogen-sufficient cells. The cells are mostly transparent. (b) Nitrogen-deficient cells. The cells are mostly opaque.

Sequences of our simultaneous fluorescence detection and image acquisition of nitrogen-sufficient and nitrogen-deficient *E*. *gracilis* cells are shown in Fig 2A and 2B, respectively. Shown in the figure panels are a splice of multiple recorded waveforms triggered by autofluorescence detection events under each culture condition. As discussed above, PMT1 detects BODIPY-tagged lipid fluorescence while PMT2 detects autofluorescence from every single cell. As shown in the figures, nitrogen-sufficient cells have a low lipid content while nitrogen-deficient cells have a high concentration of intracellular lipid. These characteristics are evident in the optical time-stretch microscope images of the cells as shown in Fig 3A and 3B. Nitrogen-sufficient cells look mostly transparent while nitrogen-deficient cells look mostly opaque throughout their entire cell body due to the high concentration of strong intracellular scatterers (presumably accumulated lipid droplets and paramylon particles). From the temporal width of the fluorescence events (~60 μs) in Fig 2A and 2B, the maximum possible throughput was found to be about 16,000 cells/s (while the actual throughput was estimated to be 10,000 cells/s as discussed above). Each image consists of approximately 720,000 pixels (approximately 200 pixels in the lateral direction and approximately 3,600 pixels in the flow direction). This excessively large number of pixels in the flow direction indicates our microscope’s ability to acquire images of cells that flow at a much higher speed. For example, cells flowing at even an extremely high speed of 25.2 m/s (corresponding to about 18 times higher throughput than 10,000 cells/s we achieved in this experiment) can be imaged by the time-stretch microscope with 200 × 200 pixels without sacrificing image quality, assuming that the microfluidic device can endure such a high normal pressure in its microchannel.

### Analysis of fluorescence events

**Fig 4:**
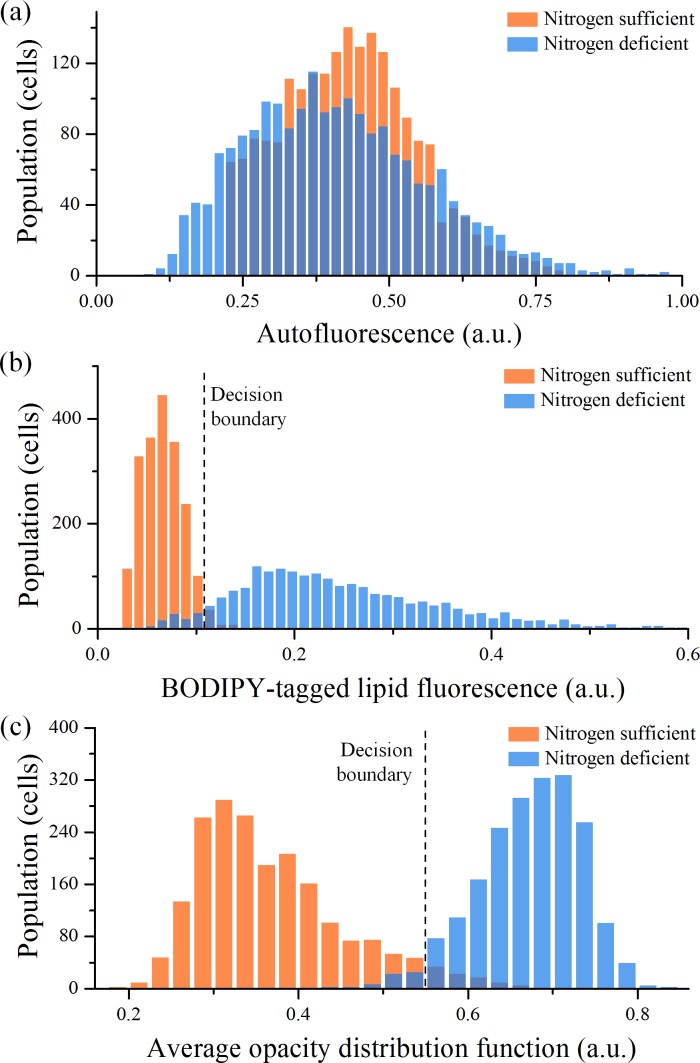
Histograms of *E*. *gracilis* cells under two different culture conditions (nitrogen sufficient and nitrogen deficient). (a) Histograms in autofluorescence signal strength (N = 2,000 for each culture). Their difference is small. (b) Histograms in BODIPY-tagged lipid fluorescence signal strength (N = 2,000 for each culture). There exists a clear difference, but a non-negligible overlap between them. (c) Histograms in average opacity distribution function value (N = 2,000 for each culture). There exists a clear difference, but a non-negligible overlap between them.

Histograms of the nitrogen-sufficient cell group (N = 2,000) and the nitrogen-deficient cell group (N = 2,000) in the autofluorescence signal strength and BODIPY-tagged lipid fluorescence signal strength are shown in Fig 4A and 4B, respectively. As Fig 4A indicates, the histogram of the nitrogen-deficient cell group was more fat and slightly shifted toward zero, resulting in a decrease in the overall intracellular chlorophyll content. On the other hand, as shown in Fig 4B, the two histograms in the BODIPY-tagged lipid fluorescence signal strength are significantly different, which means that the nitrogen-deficient cells contain more lipid than the nitrogen-sufficient cells. In the figure, a decision boundary can be drawn at a value of 0.11 (a.u.) to classify the two groups. More rigorously, the decision boundary is defined by the boundary that maximizes the accuracy defined by [30]

$$Accuracy=\frac{\sum True\;positive\;events+\sum True\;negative\;events}{Total\;population}.$$ (1)

In other words, we can use the boundary as a threshold to identify lipid-accumulated cells in an unknown heterogeneous population of *E*. *gracilis* cells, but with a false positive rate of 4.2% (calculated from 84 false positive events) or an accuracy of 95.8%. To improve the detection specificity and hence to reduce the false positive rate, an additional parameter is needed for multi-dimensional characterization of *E*. *gracilis* cells.

### Analysis of images

The image-based information provided by the optical time-stretch microscope can be combined with the fluorescence-based cell classification to reduce its false positive rate. Since, as implied by the cell images in Fig 3A and 3B, the optical transmissivity over the cell area can be used as an indicator to differentiate and classify differently cultured *E*. *gracilis* cells, we employ the opacity distribution function (ODF) [23] defined by

ODF=[O(1),O(2),⋯,O(n)], (2)

where *O*(*j*) is the opacity of the cell at pixel *j* (*j* = 1, 2, …, *n*) that ranges from 0 (completely transparent) to 1 (completely opaque) and *n* is the total number of pixels that cover the cell of interest. The average of the opacity values at all the pixels is then given by

$$aveODF=\frac{1}{n}\sum_{j=1}^{n}O(j).$$ (3)

Fig 4C shows histograms of the two cell groups in the aveODF parameter (N = 2,000 for each group). As the figure indicates, most nitrogen-sufficient cells are transparent (ranging from 0.2 to 0.7) while most nitrogen-deficient cells are opaque (ranging from 0.5 to 0.8). In this figure, just as in Fig 4B, a decision boundary can be drawn at a value of 0.55, which can be used as a threshold to identify lipid-accumulated cells with a false positive rate of 3.9% (calculated from 78 false positive events) or an accuracy of 96.1%. Here imaging plays an important role in the cell classification because the binding efficiency of fluorescent dyes to cells is known to be imperfect and fluctuating from assay to assay.

### Combined analysis of fluorescence events and images

**Fig 5:**
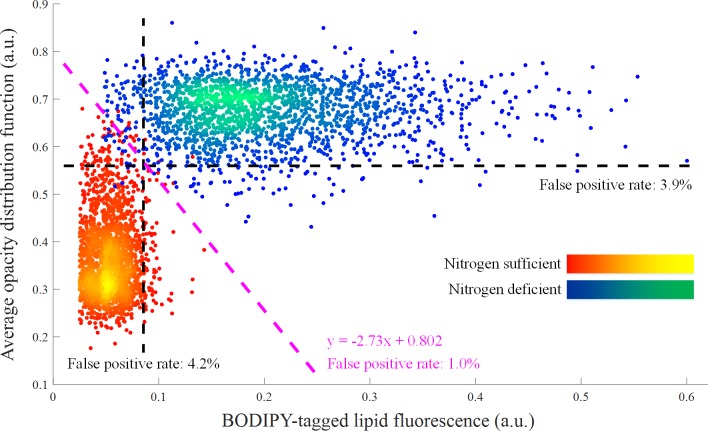
Combined scatter plot of the differently cultured E. gracilis cells in BODIPY-tagged lipid fluorescence signal strength and average opacity distribution function value (N = 2,000 for each culture). It is evident that the two culture groups can be separated more clearly with the two parameters than with either parameter alone, resulting in an improved false positive rate of 1.0%.

By combining the BODIPY-tagged lipid fluorescence signal strength and average ODF value, we can classify the groups of nitrogen-sufficient and nitrogen-deficient cells with higher accuracy. Fig 5 shows a two-dimensional scatter plot of the cell groups analyzed by the fluorescence-assisted optofluidic time-stretch microscope. As the figure indicates, each parameter’s false positive rate is about 4%, but the combined false positive rate can be as low as 1.0% (calculated from 20 false positive events over the total population of 4,000). In other words, the cell classification was made less erroneous by a factor of 4 or more accurate from about 96% to 99% by using the two screening parameters simultaneously. Here the new axis of classifying the two cell groups in Fig 5 was determined by performing linear discriminant analysis on the cells, a method used to find a linear combination of features that characterize events at a minimum possible error rate. From a practical point of view, the accurate cell classification method demonstrated here is advantageous for selective breeding of *E*. *gracilis* for efficient biofuel production [27] in which, for example, after 20 days of cultivation with every day of screening with our method, the difference in accuracy between the one-dimensional (96%) and two-dimensional (99%) screening methods becomes about 38%, which is significant enough to firmly validate the effectiveness of our powerful screening method.
